# Machine learning for psychiatry: getting doctors at the black box?

**DOI:** 10.1038/s41380-020-00931-z

**Published:** 2020-11-10

**Authors:** Dennis M. Hedderich, Simon B. Eickhoff

**Affiliations:** 1 Department of Neuroradiology, Klinikum rechts der Isar, Technical University of Munich, School of Medicine, Munich, Germany; 2 Institute of Systems Neuroscience, Medical Faculty, Heinrich Heine University Düsseldorf, Düsseldorf, Germany; 3 Institute of Neuroscience and Medicine, Brain & Behaviour (INM-7), Research Centre Jülich, Jülich, Germany

**Keywords:** Diagnostic markers, Neuroscience

## Abstract

Recent developments in the field of machine learning have spurred high hopes for diagnostic support for psychiatric patients based on brain MRI. But while technical advances are undoubtedly remarkable, the current trajectory of mostly proof-of-concept studies performed on retrospective, often repository-derived data, may not be well suited to yield a substantial impact in clinical practice. Here we review these developments and challenges, arguing for the need of stronger involvement of and input from medical doctors in order to pave the way for machine learning in clinical psychiatry.

Recent advances in algorithms and hardware have created high hopes for machine learning (ML) to become an almost universal solution for complicated problems. This enthusiasm has quickly taken over medical research, resulting in a growing number of publications highlighting the potential of ML, accompanied by increasingly strong claims to enter clinical practice [1]. With respect to brain imaging, as often a frontrunner for innovation, the motivation behind this development seems obvious: MRI is highly standardized and between-subject analyses have been established for decades. In addition, several large and open datasets provide relatively good resources for model training [2]. There is also a clinical need in psychiatry: neuropsychiatric disorders are a leading cause of morbidity and disability-adjusted life years lost worldwide, hence hopes are high for ML to accelerate the diagnostic and nosological progress in psychiatry.

Notwithstanding the potential impact of ML on psychiatry, it seems debatable whether the current state and trajectory are well aligned with the high expectations and oftentimes bold promises [3]. Especially in psychiatry, more consideration of prerequisites and further directions is needed to translate exciting proof-of-concept papers re-analyzing available datasets into clinical impact. We here highlight some of the pertinent aspects and discuss the need for increased clinical input along these steps.

While available datasets have enabled data scientists to explore many different questions, most work has focused on supervised algorithms for closed questions, in particular diagnostic classifications (“Does patient X have disease A or not?”). However, these closed-type questions are hardly reflective of clinical reality, where “open world” challenges prevail as doctors usually have to consider several differential diagnoses. These may not only have different a priori likelihoods that are again dependent on presenting symptoms and medical history, but may also coexist given the high prevalence of comorbidity, e.g. between anxiety disorders and depression or between substance abuse and psychosis. Finally, in spite of clinical complaints, a patient may actually not have any (detectable) brain disease. Consequently, even approaches showing excellent and robust performance on closed questions may be misleading in practice, if they dismiss a particular diagnosis without weighing alternative explanations. This illustrates the need for stronger consideration of actual use cases from the very start of algorithmic development. In turn, however, also expectations from the medical side need to be grounded with respect to methodological feasibility. Hence, much closer interaction between developers and users than currently practiced seems essential to avoid frustration on either side.

Driven by the idea of “AI based diagnostics”, most current research focusses on “supervised” ML, which, independent of the sophistication of architecture, in essence learns a mapping from input to target space based on a set of labeled observations (the training set). Obviously, representative training data is essential to achieve generalization to future cases, but ensuring representativeness in ML in psychiatry is challenging and extends beyond adjusting for rather obvious sociodemographic factors like sex and age. But influencing factors such as life events e.g. birth weight, obesity, or traumatic experiences, but also occupational history or child-rearing, which affect brain structure and function as well as neuropsychiatric outcomes, are widely neglected [4]. As such, they may potentially compromise ML performance through hidden stratification, which describes implications of unrecognized subsets of cases within the training set. Thus, if a depression classifier picks up on traces of childhood trauma through a high percentage of depressed patients with a history of childhood trauma, an actually depressed person without trauma may be misclassified as healthy. By training on retrospective, open-source datasets with little biographic information, these relationships get lost. Probably, the only remedy to this predicament is to increase structured reporting of biographic information in large MRI datasets and active consideration of these factors when pursuing classifiers for neuropsychiatric diseases. This calls for a close collaboration between clinicians and data scientists in order to obtain the same level of accurate and multidimensional biographic information as would be considered in clinical practice when weighting the likelihood of differential diagnoses as explanations for the current symptoms.

In addition to input features being systematically confounded by (undocumented) influences, noisy or misaligned target labels also represent a potentially serious pitfall. While this is true also for neighboring disciplines like neurology, given evolving pathophysiological concepts and diagnostic guidelines, it is a fundamental problem for psychiatry, where diagnoses are ultimately conventions on how to group symptoms into disease categories, given the lack of conclusive pathophysiological models. Hence, achieving perfect classification accuracy relative to clinical labels may actually not be desirable, particularly given that the latter are often acquired more easily in a clinical interview. On the one hand, this suggests that algorithms designed for robust learning on noisy labels may be more appropriate than those aimed at minimizing the (cross-validated) prediction error. More importantly, however, we would argue that this emphasizes the need for closed feedback-loops by clinical work-up of misclassified cases for advancing pathophysiological insight and ultimately classifications. If a healthy control was misclassified as “depressed”, can this be explained by mere technical deficiencies? Or does the subject share traits, biographic influences, even subclinical symptoms with the patients in the training group that the algorithm picked up? Such questions, which rely on more extended characterization and sufficient transparency of the algorithms, need to be addressed before visions of “precision psychiatry” can become reality. Further downstream, clinical decision makers at the deployment site need to be empowered by such knowledge in order to locally and ultimately at the individual level adapt and monitor the use of ML approaches. To ensure such ML literacy necessary for shared decision making, teaching data science to doctors needs to be developed and promoted.

As we have touched on in the previous paragraph, diagnostic labels in clinical psychiatry are notoriously unassertive with respect to their neurobiological underpinnings, limiting the usefulness of supervised ML strategies. As an alternative not relying on labeled data, unsupervised learning groups individuals based on detected patterns in high-dimensional data [5]. Once robust patterns are established, these may then inform pathophysiological concepts in psychiatry by comparison to clinical (phenomenologically driven) nosology or individual psychopathological work-up of the patients as described above for misclassified cases. Such approach would resonate well with the Research Domain Criteria (RDoC) idea of dimensional psychiatry, and the increasing popularity of canonical correlation analysis (CCA) finding linked components in imaging and clinical data. Together, these may then lead to important refinements of current concepts for psychiatric disease classification. However, we would like to note that unsupervised approaches will inevitably find components in the data, which does not make these by themselves useful in clinical practice. But any ML approach in the diagnostic work-up of patients in psychiatry will only succeed if it creates an impact in real life, e.g. through choosing the most beneficent therapeutic option for a patient. This being said, it becomes obvious that the prognostication of treatment response may even be a more relevant question for ML to solve in psychiatry than correctly assigning a diagnostic label. Again, the evaluation of this impact needs to thoroughly involve medical experts as it entails multiple facets beyond, e.g. factor stability or generalization. On the one hand, it may promise more appropriate interventions, better long-term outcome and reduced socio-economic costs, but conversely also deteriorating patient-physician relationship, unclear accountability and difficult acceptance [6]. Critically weighting benefits and drawbacks of ML in psychiatry calls for prospective, multi-center designs in a realistic clinical environment as opposed to the currently prevailing proof-of-concept studies. Learning from other fields like pharmacological drug testing, where the introduction of preregistration and external monitoring dramatically reduced the number of positive studies, this will likely lead to sobering results [7]. There is also a pertinent rationale for a closer integration of clinical (drug) trials and ML in psychiatry, especially for the prediction of treatment outcomes. Neuropsychopharmacology represents the main therapeutic option for most psychiatric disorders but non-response rates are high. Integrating ML into clinical trials could thus open new opportunities towards a better insight into patient- or setting-specific factors that may influence the therapeutic response, and ultimately allow more targeted deployment of (new) drugs. Such models towards precision psychiatry need to be validated themselves in separate trials and ML-suggested stratifications need to be tested for their added benefit over clinical best-practice recommendations for the choice of therapeutic agents. In this context, we also note that realization of such potential will largely depend on the willingness and ability of psychiatrists and healthcare providers to integrate such novel markers into clinical routine; a non-trivial task for psychiatrists outside academic institutions, which in many countries provide the majority of care. Will such extensive evaluation protocols slow down technical innovation in a fast-moving field? Most likely, but if ML for medical imaging aspires to have an impact similar to pharmaceutical treatment, it seems indispensable to hold its evaluation to similar standards. This is even more true when dealing with a yet poorly understood and complex organ as the brain in the context of multidimensional concepts of neuropsychiatric diseases.

In summary, we argue for a deeper involvement of domain experts, particularly medical professionals, in the process of developing novel ML applications for clinical psychiatry to help fulfill the currently high expectations.
